# Effects of a 7-day step taper, training cessation, and retraining on lower body physical and morphological aspects in elite female ice hockey players

**DOI:** 10.3389/fspor.2025.1541765

**Published:** 2025-07-01

**Authors:** Daichi Nishiumi, Daichi Yamashita, Takanori Kurokawa, Naoki Hirase, Kazuki Wakamiya, Iñigo Mujika, Norikazu Hirose

**Affiliations:** ^1^ Faculty of Sport Sciences, Waseda University, Saitama, Japan; ^2^ Department of Sport Science and Research, Japan Institute of Sports Sciences, Tokyo, Japan; ^3^ Graduate School of Sport Sciences, Waseda University, Saitama, Japan; ^4^ Department of Physiology, Faculty of Medicine and Nursing, University of the Basque Country, Leioa, Basque Country; ^5^ Exercise Science Laboratory, School of Kinesiology, Faculty of Medicine, Universidad Finis Terrae, Santiago, Chile

**Keywords:** braking phase force, rate of force development, vertical jump performance, force-velocity profile, muscle architecture

## Abstract

**Purpose:**

This study investigated the effects of a 7-day step taper before a national championship, followed by 2 weeks of training cessation and 2 weeks of retraining on lower body physical and morphological aspects.

**Methods:**

Seven elite female ice hockey players (age: 23 ± 4 years) participated in six testing sessions: two baseline sessions, one pre-championship (after taper), one after a 2-week training cessation, and two following retraining (after 1 week and after 2 weeks). Assessments included body composition, vastus lateralis muscle architecture (muscle thickness, pennation angle, fascicle length), and lower body vertical force–time metrics [countermovement jump (CMJ), squat jump (SJ), and loaded SJ]. During the tapering period, training duration was reduced by 35%. During retraining, participants performed three weekly sessions of fast eccentric squats, with 4 sets of 8 repetitions per session, gradually increasing intensity from 45.5% to 63.5% of estimated 1RM.

**Results:**

Tapering did not affect any of the lower body physical and morphological measures. Training cessation induced declines in braking rate of force development (13.1%, *p* < 0.05), braking peak force during CMJ (5.5%, *p* < 0.05), and muscle mass (3.6%, *p* = 0.03). All metrics returned to post-taper values within 1 week of retraining.

**Conclusions:**

2 weeks of training cessation induced minor declines in eccentric muscle performance, but recovered within 1 week of retraining. This study provides valuable insights for practitioners in designing effective peaking, off season and retraining programs in female ice hockey.

## 1 Introduction

Ice hockey is a sport that demands repeated high-intensity sprints and rapid change of direction maneuvers on the ice (1). The typical format of an ice hockey season within the top-tier domestic teams in Japan encompasses national league (9 matches) and local league (4 matches), followed by a national championship (1–4 matches) for a total of 14–17 matches over 6 months. As the season progresses towards the playoffs, players usually undergo short-term (approximately 1–2 weeks) tapering to peak for the national championship, after which training usually ceases for 2–4 weeks (off-season). Subsequently, players begin retraining in preparation for the upcoming season. Unlike North American league (25–30 matches) (2), the competitive season for Japanese female ice hockey players consists of fewer matches spread across a longer period, often followed by an off-season. Therefore, understanding the effects of tapering, training cessation, and retraining is crucial for long-term progression in this specific population.

The ability to skate quickly to gain puck possession and/or reach a high velocity to challenge the opposing team are major determinants of ice hockey performance (1). Therefore, besides skating skills, the ability to rapidly accelerate and decelerate the body, which is underpinned by substantial lower body muscular strength and power, is vital. To enhance these physical qualities, most ice hockey strength and conditioning coaches prescribe periodized programs of on- and off-ice training sessions, as well as off-ice testing (3). Furthermore, strength and power are typically developed progressively from the pre-season through the regular season, maintained or further enhanced during the tapering phase, and then re-developed during the retraining phase following the off-season. On-ice sprint performance was found to correlate with off-ice maximal strength performance (4) and countermovement jump (CMJ) performance (4, 5). Specifically, professional male ice hockey players exhibited superior abilities in generating ground reaction force (GRF) during CMJ compared to Under-20 counterparts within the same professional organization; on-ice sprinting speed correlated with superior CMJ performance, but not squat jump (SJ) performance (6). Furthermore, the lower limbs undergo eccentric loading during on-ice change of direction (COD) maneuvers (7). This is relevant as eccentric braking capacity during the CMJ has been linked to superior on-ice COD performance (8), highlighting the potential benefits of enhancing lower limb explosive performance for improved on-ice performance. Moreover, the vertical force-velocity profile (FVP) (9) and reactive strength index-modified (RSImod; jump height divided by movement duration) (10), both derived from jump tests, are indicators of explosive capacity in the lower limbs, and resistance training can enhance this capacity (11). Muscle morphology can also influence lower body power output. Notably, strength training can induce changes in muscle architecture, including increased fascicle length and muscle thickness, alongside a decreased pennation angle (12).

Numerous studies have shown that short-term peaking for major competitions can significantly improve lower body power (13), muscle architecture (12), and FVP (14) in team-sport athletes. On the other hand, 4 weeks of training cessation during the off-season can lead to changes in muscle strength, power (15) and muscle architecture (12). However, a notable gap remains regarding the effects of short-term training cessation after peaking, and subsequent retraining, particularly in female team-sport athletes. There is some evidence showing a decline in physical performance after a short (i.e., two weeks) training cessation. Yamashita et al. (16) reported that highly trained sprinters did not experience a decrease in knee concentric muscle strength (60 deg/s), but they did experience a significant decrease in eccentric muscle strength. Similarly, Hortobágyi et al. (17) observed a decrease in eccentric muscle strength during knee joint extension. These results suggest that eccentric capacity, which is critical for on-ice performance, tends to decline early following training cessation and should be specifically targeted during the pre-season retraining period. A previous study has reported that even low-volume squats, when performed with an emphasis on the speed of the eccentric phase (i.e., the lowering phase), can effectively enhance rate of force development (RFD) (18). Furthermore, Mujika et al. (19) reported that athletes who demonstrate a smaller decline in performance during the off-season tend to achieve a better peak performance during the subsequent season. This suggests that minimizing the decline in physical abilities during the off-season is crucial for achieving better performance in the subsequent season. It could be speculated that changes in eccentric capacity during tapering, training cessation, and retraining could impact an athlete's individual skating performance, as well as overall team performance for long-term progression.

Therefore, the aim of this study was to investigate the effects of short-term tapering, training cessation and retraining on body composition and lower body physical and morphological aspects (i.e., muscle architecture, and vertical force–time metrics during jump tests) in female ice hockey athletes. The hypotheses are as follows: (1) during the tapering period, an improvement in fitness indicators is expected, (2) during the short-term training cessation period, there might not be significant changes in power, concentric RFD, or jump height, but be a potential decline in eccentric performance, (3) during the retraining period, a recovery in the diminished eccentric capacity is anticipated.

## 2 Materials and methods

### 2.1 Participants

The G*Power version 3.1.9.2 (Heinrich-Heine-Universität Düsseldorf, Düsseldorf, Germany) was used to determine the appropriate sample size for this study. For a repeated-measures analysis of variance (ANOVA), we set the power at 80%, alpha at 5%, and an effect size f of 0.436 (partial *η*^2^ = 0.16), requiring a minimum of eight participants. We initially recruited nine elite-level (20) female ice hockey players from a club team, without musculoskeletal disorders. Two athletes were excluded due to scheduling conflicts, leaving seven participants (23 ± 4 years, 158.7 ± 5.7 cm). Five participants had prior experience on the World University Championship team, one on the U18 national team, and all had over one year of resistance training experience. A sensitivity analysis determined the required effect size f to be 0.455 (partial *η*^2^ = 0.18), used as a threshold for interpreting our findings. This study was approved by the Institutional Review Board (No. 2021-069) and conducted in accordance with the Declaration of Helsinki. Participants received comprehensive verbal and written information about potential risks and provided informed consent.

### 2.2 Procedures

**Figure 1:**
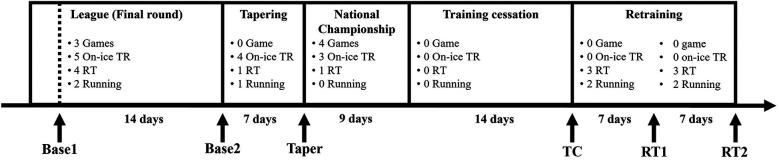
Schedule and timing of tests during the experimental period. Abbreviations: TR, training; RT, resistance training; Base 1, first baseline test; Base 2, second baseline test, TC, test after two-week training cessation; RT1, test after one-week retraining; RT2, test after two-week retraining. Note: One resistance training session was conducted before the national championship tournament; however, it was a self-selected session, and individual details were not recorded.

This study involved six testing sessions: two baseline sessions (Base 1 and Base 2), one before the national championship tournament (Taper), one after a 2-week training cessation (TC), and two following retraining (after 1 week: RT1, and after 2 weeks: RT2) (Figure 1). To eliminate the influence of acute fatigue, all tests were performed before any scheduled training sessions on testing days. Testing sessions followed a consistent order: body composition, muscle architecture, and jump tests. During the training cessation period, participants were instructed not to engage in any lower body training exercises and were also advised against the consumption of supplements. The retraining phase included three resistance training sessions and two running sessions per week.

### 2.3 Data analysis

Body composition was measured using the bioelectrical impedance analysis (InBody 770, InBody Japan Inc., Tokyo, Japan), assessing body mass, lean body mass, body fat mass, body fat percentage, and lower body muscle mass. Muscle architecture was measured using ultrasound (UITC-640A, Viamo, Canon Medical Systems Inc., Tochigi, Japan), evaluating the muscle thickness, pennation angle, and muscle fascicle length of the vastus lateralis. In a standing position, the ultrasound probe (6.5 cm) was placed along the longitudinal axis of the vastus lateralis, targeting the midpoint between the lateral epicondyle and the superior-lateral border of the patella. Three images were captured for reliability. All ultrasound measurements were conducted by the same examiner to maintain consistency. Prior to data collection, the examiner underwent extensive familiarization with the ultrasound procedures and image acquisition techniques. Muscle thickness and pennation angle were measured using image analysis software (ImageJ, National Institutes of Health, Maryland, USA), and fascicle length was calculated using the following validated formula (21).

Fasciclelength=musclethickness/sin(pennationangle)

Using a wireless dual force platform system (1,000 Hz, Hawkin Dynamics, Westbrook, Maine, USA), two trials of each jump type (bodyweight CMJ with a 0.5 kg bar, bodyweight SJ, and loaded Smith machine SJ) were performed in that order. Participants had a rest interval of 1–3 min between bodyweight jump trials, and 2–4 min between loaded jumps. Prior to the jump tests, participants underwent a warm-up consisting of 5 min running and 5 min dynamic stretching. Then, five submaximal bodyweight jumps (either CMJ or SJ) were completed for each specific jump test.

The countermovement depth in the CMJ and the starting position for the SJs were standardized to a 90-degree knee angle. Since different countermovement depths in the CMJ can affect variables related to the braking phase (22), the countermovement depth was standardized across all testing sessions. Furthermore, the participants regularly engaged in jump training and were accustomed to performing CMJs to an approximate 90-degree knee flexion. A timing gate system (VoltOnoSprint, S-CADE Inc., Tokyo, Japan) was positioned beneath the participants to ensure consistency, emitting a beep when the buttocks interrupted the sensor. The participants were instructed to “maintain a quiet standing position for more than 1 s and then execute a rapid countermovement as fast as possible and jump as fast as possible” for the CMJ and “maintain a starting position for more than 1 s and then jump as fast as possible without using any countermovement” for the SJ. If the countermovement depth did not reach the pre-set height (confirmed by a beep), or if any countermovement was detected at the start of the SJ (indicated by a decrease in vertical ground reaction force by 5%), the attempt was deemed a failure, and the jump was repeated. The following parameters were obtained from the software: jump height (from impulse-momentum method), braking RFD, braking peak force and RSImod for the CMJ, and jump height, concentric RFD, and concentric peak force for the bodyweight SJ, and jump height for the loaded SJ. The average values were used for the analysis.

The FVP was estimated using a 2-point method involving bodyweight SJ and loaded Smith machine SJ (23). The loaded Smith machine SJ were performed with 75% of body mass. Following Samozino's method (9), push-off height was calculated by subtracting the vertical distance from the greater trochanter to the ground during a SJ with 90 degrees of knee flexion from the length between the greater trochanter and the tip of the toes measured in the supine position with full extension of the hip, knee, and ankle joints. These heights, along with jump height and the system mass for both bodyweight and loaded SJ, were entered into Samozino's Excel file to calculate the FVP. From these values, the force-velocity profile parameters *F*_0_ (force intercept), *V*_0_ (velocity intercept), and *P*_max_ (peak power) were calculated.

### 2.4 Periodization of training and load management

The intensity of the on-ice sessions could not be quantified, but on-ice practice lasted approximately 90 min and ice hockey games lasted approximately 120 min. The duration of resistance training was 70 min during league period, 60 min during tapering, and 20 min during retraining, respectively. Therefore, the training duration was 9.75 h/week during the league period, 6.33 h/week during tapering, 10.5 h/week during the national championship tournament including 4 games, and 2.3 h/week during the retraining period. Therefore, the total training time during the tapering period was approximately 35% less than the league period. The intensity for main exercises during the tapering period was unchanged compared to the previous week (87.5% one-repetition maximum; 1RM) (Table 1). During the training cessation period, lower body training was prohibited, along with the consumption of supplements, and the athletes were allowed to engage in their normal daily activities. During the retraining period, training intensity gradually increased from 45.5% to 63.5% 1RM (Table 2).

**Table 1:** Resistance training programs for baseline and tapering periods.

Baseline (Base 1 and Base 2)				Tapering			
Exercises	Load	Sets	Reps	Exercises	Load	Sets	Reps
Deadlift	87.5% 1RM	3	4	Jump squat	30% 1RM (max effort)	3	4
Assisted jump	–	3	5	Back squat	87.5% 1RM	2	5
Bench press	87.5% 1RM	3	5	Plyometric push-up	Bodyweight	3	4
Resisted sprint (10 m)	–	1	7	Prone Bench pull	3RM	3	3
Loaded pull up	3RM	3	3	Sprint (10 m)	–	1	7
One leg squat	Bodyweight	2	8				

Abbreviations: Base 1 and Base 2; first and second baseline tests (final round of the league tournament); 1RM, 1-repetition maximum.

**Table 2:** Off-ice training program during the retraining period.

Exercise volume and intensity of the retraining period						
Week 1	Sessions	Session 1	Session 2	Session 3	Session 4	Session 5
	Exercise	FESQ	Jog	FESQ	Jog	FESQ
	Intensity	45.5% 1RM	140–150 bpm	50.0% 1RM	140–150 bpm	54.5% 1RM
	Volume	4 sets × 8 reps	40 min	4 sets × 8 reps	40 min	4 sets × 8 reps
Week 2	Sessions	Session 6	Session 7	Session 8	Session 9	Session 10
	Exercise	FESQ	Jog	FESQ	Jog	FESQ
	Intensity	59.0%1RM	140–150 bpm	63.5%1RM	140–150 bpm	63.5%1RM
	Volume	4 sets × 8 reps	40 min	4 sets × 8 reps	40 min	4 sets × 8 reps

Abbreviations: FESQ, fast eccentric squat; bpm, beats per minute; 1RM, one-repetition maximum.

The retraining sessions spanned 2 weeks, comprising ten sessions in total, with three fast eccentric squat (FESQ) sessions and two running sessions per week. The running sessions occurred on separate days from the FESQ sessions, lasting 40 min while maintaining a heart rate between 140 and 150 bpm (T037, EZON Inc., China). Each resistance training session began with static and dynamic stretching, followed by a warm-up consisting of five squat repetitions with a chosen weight. The FESQ was performed to a metronome, emphasizing lowering as fast as possible and taking 3 s for the ascending phase. Feet were shoulder-width apart with toes slightly outward, and the barbell was on the shoulders. The depth was set to half squat (knee joint at 90 degrees). Each session included 4 sets of 8 repetitions.

To safely determine the intensity of FESQ, an estimated 1RM in the half squat was calculated from the 2-point FVP. First, the 1RM for the SJ (a theoretical load at which take-off is no longer possible) was determined using the FVP (24). Then, the estimated 1RM for the half squat was calculated based on a previous study comparing the estimated 1RM of SJ and half squat (136 kg vs. 150 kg, respectively) (24). The intensity gradually increased from approximately 45.5%–63.5% of the estimated 1RM. The intensity of the FESQ was set at 63.5% 1RM, reflecting the 60%–80% 1RM range maximizing descending-phase RFD (18). However, considering the participants' detraining, it was believed that applying a high load would be too strenuous. The intensity was gradually increased by 4.5% for each FESQ session starting from 45.5% 1RM (Table 2).

### 2.5 Statistical analysis

Statistical analyses were conducted using SPSS version 28 (IBM, Armonk, NY, USA). A significance level of *p* < 0.05 was used. Normality was assessed using the Shapiro–Wilk test. The data are reported as mean ± standard deviation (SD) for normally distributed variables and as median and interquartile range for non-normally distributed variables.

The inter-day (Base 1 and Base 2) reliability was assessed using the intraclass correlation coefficient (ICC_2,2_) or concordance correlation coefficient (CCC) for non-normally distributed. The coefficient of variation (CV) was also calculated. Acceptable reliability was defined as an ICC or CCC > 0.75 and a CV < 10%.

To examine the effects of tapering, detraining and retraining, a one-way repeated measures ANOVA was conducted in the Base 2, Taper, TC, RT1, and RT2. For non-normally distributed data, a Friedman test was performed. Pairwise comparisons used the Bonferroni *post-hoc* test. The effect sizes were calculated using partial eta-squared (*η*^2^) for ANOVA, Hedges's *g* for pairwise comparisons, and *r* for the Friedman test, interpreted as small: >0.01, moderate: >0.06, and large: >0.138 for partial *η*^2^ and small: >0.2, medium: >0.5, large: >0.8 for both *g* and *r* (25)*.*

## 3 Results

The inter-day reliabilities for all variables, except for *V*_0_ satisfied the criteria for ICC or CCC (>0.75) and CV (<10%), demonstrating high reliability (Table 3).

**Table 3:** Inter-day reliability of the variables between the first baseline test (Base 1) and the second baseline test (Base 2).

Measured parameters	Variables	ICC (95%CI)	CCC (95%CI)	CV (%)
Body compositions	Body mass (kg)	0.99 (0.98–1.00)		0.6 ± 0.6
	Lean body mass (kg)	0.92 (0.63–0.99)		1.7 ± 2.5
	Lower-body muscle mass (kg)	0.98 (0.89–1.00)		1.6 ± 0.9
	Body fat percentage (%)	0.91 (0.57–0.99)		4.5 ± 3.8
CMJ	Height (m)	0.94 (0.62–0.99)		2.9 ± 2.9
	Braking RFD (N/s)	0.95 (0.71–0.9)		4.5 ± 3.4
	Braking peak force (N)		0.94 (0.68–1.00)	1.7 ± 1.7
	RSImod		0.94 (0.69–1.00)	2.6 ± 1.5
SJ	Height (m)	0.94 (0.69–0.99)		2.4 ± 2.6
	Concentric RFD (N/s)	0.87 (0.33–0.98)		6.5 ± 6.7
	Peak force (N)	0.99 (0.95–1.00)		1.5 ± 1.1
FVP	*F*_0_/BM (N/kg)	0.91 (0.45–0.98)		5.2 ± 4.2
	*V*_0_ (m/s)	0.09 (−0.84–0.85)		6.4 ± 6.9
	*P*_max_ (W)	0.96 (0.79–0.99)		1.9 ± 2.3
Muscle architecture (vastus lateralis)	Muscle thickness (cm)	0.95 (0.50–0.99)		5.0 ± 1.6
	Pennation angle (°)		0.84 (0.52–1.00)	4.9 ± 4.0
	Fascicle length (cm)		0.77 (0.38–1.00)	3.9 ± 5.3

Abbreviations: CMJ, countermovement jump; SJ, squat jump; FVP, force-velocity profile, Braking RFD, rate of force development during braking phase; RSImod, reactive strength index-modified; Concentric RFD, rate of force development during concentric phase; BM, body mass; *F*_0_, theoretical maximum force; *V*_0_, theoretical maximum velocity; *P*_max_, maximal power; ICC, intra-class correlation coefficient; CI, confidence interval; CCC, concordance correlation coefficient; CV, coefficient of variation. Note: CV values are presented as mean ± SD.

For body composition, a one-way ANOVA revealed that lean body mass and lower body muscle mass significantly decreased during the 2-week training cessation (3.6%, *p* = 0.03, 3.0%, *p* = 0.03, respectively), but returned to normal after a one-week retraining phase (Table 4). No significant changes were observed in the other variables throughout the follow-up.

**Table 4:** Changes in body composition metrics throughout a competitive season and off-season span.

Body compositions	Base 1	Base 2	Taper	TC	RT1	RT2	*p*	Partial *η*^2^
Body mass (kg)	58.3 ± 6.5	58.5 ± 6.3	58.9 ± 6.4	58.2 ± 6.2	58.4 ± 6.3	58.2 ± 6.7	0.12	0.25
Lean body mass (kg)	43.7 ± 4.3	44.5 ± 4.0	44.9 ± 4.4*^,†^	43.3 ± 4.1*	43.8 ± 4.5	43.2 ± 4.1^†^	<0.01	0.58
Lower-body muscle mass (kg)	13.1 ± 1.5	13.1 ± 1.6	13.5 ± 1.6*	13.1 ± 1.5*	13.3 ± 1.5	13.3 ± 1.6	<0.01	0.43
Body fat percentage (%)	23.5 ± 4.9	23.6 ± 4.0	23.6 ± 4.2	25.4 ± 3.9	24.9 ± 3.6	25.5 ± 4.3	<0.01	0.43

Abbreviations: Base 1, first baseline test; Base 2, second baseline test; TC; test after two-week training cessation; RT1, test after one-week retraining; RT2, test after two-week retraining.

The values are presented as mean ± SD.

Notes: *: Significant difference between Taper and TC.

^†^ Significant difference between Taper and RT2.

Regarding muscle architecture, one-way ANOVA revealed significant main effects in fascicle length (*p* = 0.045, *r* = 0.57). However, no significant differences were observed for muscle thickness and pennation angle.

**Figure 2:**
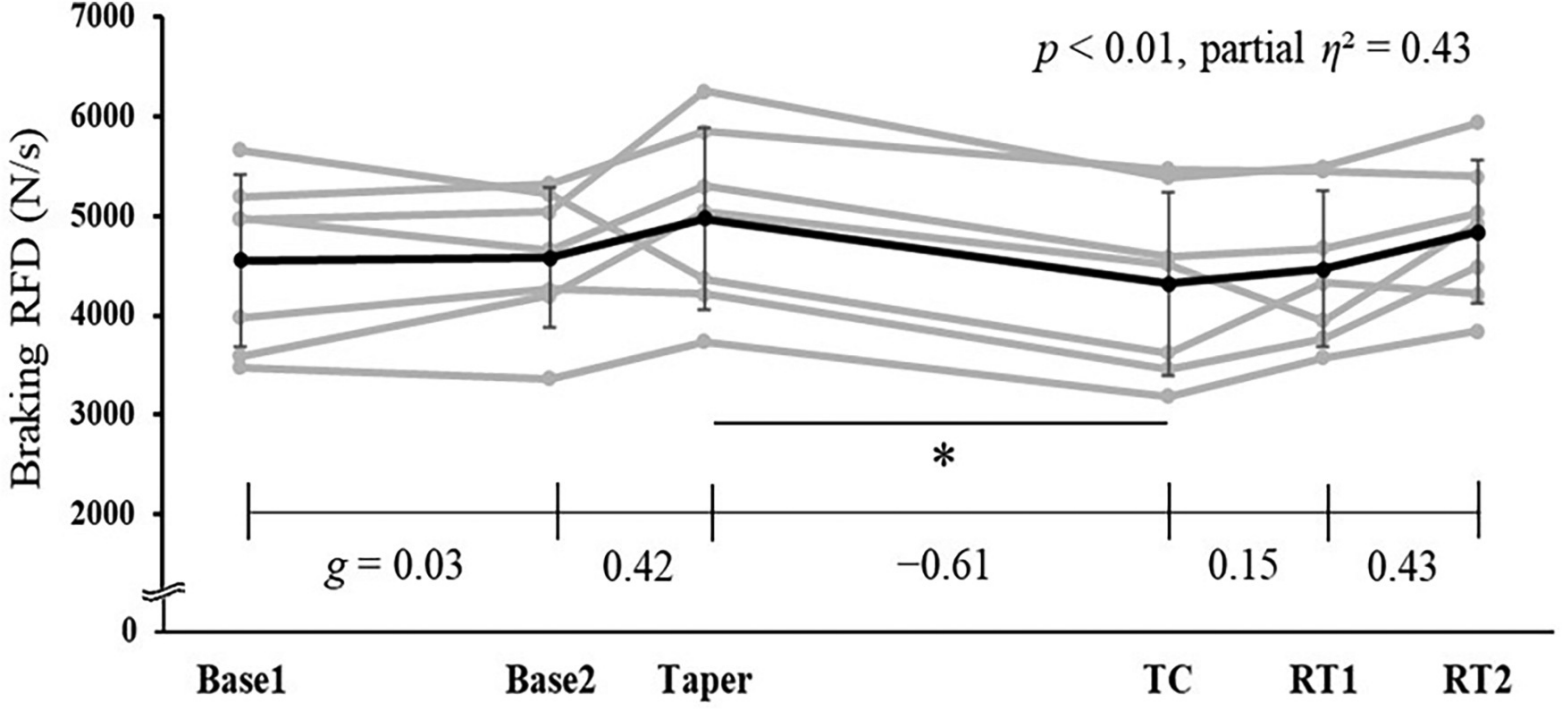
Changes in braking rate of force development (RFD) during countermovement jump throughout a competitive season and off-season span. Abbreviations: Base 1, first baseline test; Base 2, second baseline test, TC, test after two-week training cessation; RT1, test after one-week retraining; RT2, test after two-week retraining. *p < 0.05.

**Figure 3:**
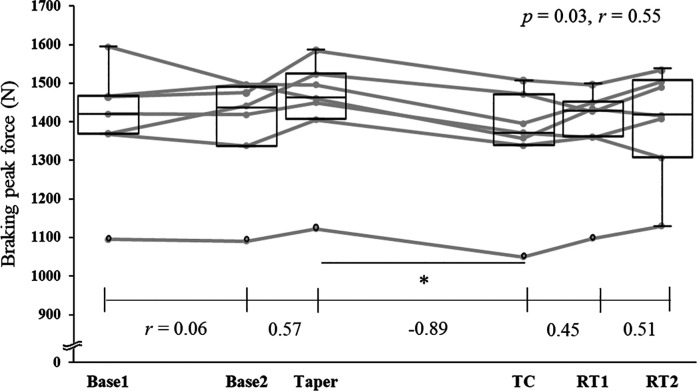
Changes in braking peak force during countermovement jump throughout a competitive season and off-season span. Abbreviations: Base 1, first baseline test; Base 2, second baseline test, TC, test after two-week training cessation; RT1, test after one-week retraining; RT2, test after two-week retraining. Notes: Individual data points are shown with lines connecting the corresponding values across the phases. Box plots display the interquartile range (IQR), median, and whiskers indicating the highest and lowest data points. When outliers are present (i.e., when the data points fall outside the range of 1.5 × IQR), the higher/lower whisker represents the highest/lowest data point excluding the outliers. *p < 0.05.

For jump performance, One-way ANOVA and Friedman test indicated significant main effects in braking RFD (*p* < 0.01, partial *η*^2^ = 0.43), braking peak force (*p* = 0.03, *r* = 0.55), concentric RFD (*p* = 0.02, *r* = 0.57), and concentric peak force (*p* = 0.01, partial *η*^2^ = 0.40) (Figures 2, 3, Table 5). *Post-hoc* tests revealed significant decrease between Taper and TC in both braking RFD (13.1%, *p* < 0.01) and braking peak force (5.5%, *p* = 0.04). No significant changes were observed between Taper and TC in jump height for CMJ and SJ, concentric RFD, and concentric peak force. After retraining, the braking RFD and braking peak force recovered within one week. The 2-week training cessation resulted in the maintenance of the RSImod and FVP.

**Table 5:** Changes in body physical and morphological aspects throughout a competitive season and off-season span.

Measured parameters	Variables	Base 1	Base 2	Taper	TC	RT1	RT2	*p*	Partial *η*^2^	*r*
CMJ	Jump height (m)	0.335 ± 0.041	0.334 ± 0.034	0.340 ± 0.037	0.327 ± 0.045	0.336 ± 0.038	0.339 ± 0.040	0.42	0.15	
	RSImod	0.43 ± 0.06	0.43 ± 0.05	0.44 ± 0.05	0.42 ± 0.08	0.43 ± 0.05	0.44 ± 0.05	0.40		0.34
	Countermovement depth (m)	0.379 ± 0.024	0.371 ± 0.032	0.376 ± 0.025	0.371 ± 0.035	0.365 ± 0.022	0.356 ± 0.018	0.13		0.38
SJ	Jump height (m)	0.301 ± 0.034	0.297 ± 0.027	0.301 ± 0.042	0.290 ± 0.038	0.295 ± 0.029	0.295 ± 0.036	0.64	0.10	
	Concentric RFD (*N*/s)	1,806.8 ± 335.3	1,955.8 ± 414.2	1,948.1 ± 378.1^‡^	1,848.3 ± 434.3	2,128.4 ± 264.1	2,197.4 ± 489.1^‡^	0.02		0.57
	Concentric peak force (*N*)	1,251.1 ± 158.6	1,262.9 ± 168.7	1,285.8 ± 169.8	1,278.9 ± 164.3	1,309.4 ± 171.9	1,317.4 ± 182.2	0.01	0.40	
FVP	*F*_0_/BM (*N*/kg)	36.4 ± 4.6	35.9 ± 5.7	35.9 ± 4.9	35.4 ± 4.9	36.0 ± 3.2	37.2 ± 4.1	0.74	0.08	
	*V*_0_ (m/s)	2.29 ± 0.19	2.36 ± 0.28	2.36 ± 0.37	2.36 ± 0.33	2.32 ± 0.16	2.27 ± 0.27	0.75		0.24
	*P*_max_/BM (W/kg)	20.7 ± 2.17	20.8 ± 2.46	20.9 ± 2.5	20.5 ± 2.3	20.8 ± 1.5	21.0 ± 2.4	0.9	0.04	
Muscle architecture (vastus lateralis)	Muscle thickness (cm)	2.41 ± 0.42	2.54 ± 0.36	2.44 ± 0.32	2.79 ± 0.54	2.78 ± 0.53	2.77 ± 0.59	0.4		0.33
	Pennation angle (°)	19.5 ± 3.4	20.8 ± 3.3	21.1 ± 2.3	19.8 ± 2.3	20.3 ± 2.0	21.0 ± 2.3	0.07	0.33	
	Fascicle length (cm)	7.34 ± 1.48	7.19 ± 0.75	6.78 ± 0.66^†^	8.64 ± 3.37	8.2 ± 2.4^†^	7.9 ± 2.5	0.045		0.57

Abbreviations: CMJ, countermovement jump; SJ, squat jump; FVP, force-velocity profile; RSImod, reactive strength index-modified; Concentric RFD, rate of force development during concentric phase; BM, body mass; *F*_0_, theoretical maximum force; *V*_0_, theoretical maximum velocity; *P*_max_, maximal power; Base 1, first baseline test; Base 2, second baseline test; TC, test after two-week training cessation; RT1, test after one-week retraining; RT2, test after two-week retraining; CV, coefficient of variation.

The values are presented as mean ± SD.

^†^ Significant difference between Taper and RT1.

^‡^ Significant difference between Taper and RT2.

## 4 Discussion

This study investigated the effects of short-term tapering, training cessation and retraining on lower body physical and morphological aspects in female ice hockey athletes. The main findings of this study were: (1) during the 7-day tapering period, there were no significant changes in any lower body physical and morphological measures; (2) during a 2-week training cessation, a significant decrease was observed in braking RFD and braking peak force during the CMJ, but there were no changes in jump height during the CMJ and SJ; (3) during the first week of retraining, the measures recovered towards post-taper values.

Contrary to our first hypothesis, lower body physical and morphological aspects did not improve during tapering. Previous studies suggested short-term tapering for major competitions improve lower body power (13, 26), muscle architecture (12), and FVP (14) in team sports. However, a competitive season can induce fatigue accumulation and performance degradation. Gannon et al. reported that a professional ice hockey season decreased CMJ performance, with the effects of fatigue most prominent during the late-season phase (27). Bazyler et al. (28) reported that tapering between the regular season and conference championships maintained jump performance, but decreased muscle thickness in female collegiate volleyball players. In the present study, tapering was conducted in the late-season phase for the national championship tournament, and physical performance was adequately maintained, but not improved. It is possible that a 7-day taper and a decreased training duration of 35% may not allow sufficient recovery for performance peaking. Indeed, a 2-week tapering with a 41%–60% reduction in training volume has been suggested as the most effective strategy for maximizing performance gains (29).

In partial agreement with our second hypothesis, the braking RFD and braking peak force significantly decreased following a 2-week training cessation, although no changes were observed in jump height, concentric force values, RSImod, and FVP. This indicates that although ice hockey players may maintain their ability to accelerate rapidly, as well as their maximum strength and power, a 2-week training cessation may lead to a decline in their ability to decelerate rapidly. Previous studies have also reported a decline in eccentric muscle torque (16, 17) and Type II muscle fibers (17) as a consequence of a 2-week training cessation, which is similar to the findings of this study. Furthermore, since deceleration ability contributes not only to performance enhancement but also to sports injury prevention (30), these findings can be highly valuable in the reconditioning process for returning to competition after sports injuries.

Three possible factors could explain the decrease in braking RFD and peak force. First, the contraction pattern during the braking phase of the CMJ involves eccentric or quasi-isometric contractions, resisting muscle elongation due to external forces (31). Therefore, a 2-week training cessation likely decreases eccentric muscle strength, negatively impacting force production during the braking phase. Second, a potential decrease in Type II muscle fibers may contribute to the decline in braking RFD and peak force. Although muscle fiber composition was not measured in the present study, the observed decrease in lean body mass and lower limb muscle mass following the two-week training cessation may indicate a potential decrease in Type II muscle fibers, as suggested by Hortobágyi et al. (17). During the eccentric phase, the principle of muscle size is disrupted, and Type II fibers are preferentially recruited (32); therefore a reduction in Type II fibers is likely to have a greater impact on eccentric muscle strength than on concentric strength. Third, the muscle-tendon unit (MTU) interaction during deceleration may change. Tendon elongation without significant muscle elongation increases peak GRF (33), aiding rapid deceleration. Research on the repeated bout effect suggests that during the second eccentric training session, muscles are less stretched, and tendons elongate more compared to the first session (34). Training cessation might alter MTU interactions, making muscles less quasi-isometric and reducing peak GRF. In our study, the CMJ test, involving rapid joint angular velocities and MTU interaction, suggests possible decreased force production during the braking phase. However, as these factors were not directly measured, definitive conclusions cannot be drawn. Future research directly investigating these factors is needed for clearer understanding.

The GRF during the braking phase showed significant recovery within one week of retraining (3 sessions of FESQ at about 50% 1RM for 20 min per session). FESQ, performed by squatting as fast as possible with the load, creates a rapid and large external load during deceleration, exposing muscles to stress and rapidly stretching them with high tension. This likely stimulates Type II fibers and improves MTU interaction, contributing to increased eccentric and quasi-isometric muscle strength. FESQ is known to improve braking RFD and Type II fiber cross-sectional area (18). Therefore, the braking phase GRF may have recovered within one week of retraining. Such exercises may be beneficial for returning to play after a short-term training cessation. However, whether similar results can be achieved with other training interventions remains uncertain and warrants further investigation.

As a limitation of this study, we were unable to evaluate menstrual cycle variations, despite inconclusive findings related to physical performance (35). Furthermore, as this study spans multiple periodization phases over more than two months, it is impractical to delineate the influence of menstrual cycle variations on each contributing factor. Since group averages are used in our analyses, the potential impact of individual menstrual cycle variations is likely minimized within these averages. Another limitation is the potential influence of diurnal variation. Because some of the measurements were conducted during the competitive season, the team's schedule varied, making it difficult to perform all measurements at the same time of day. Therefore, caution is warranted when interpreting the results. Although diurnal variations in jump performance have been shown to be greater when using a preferred countermovement depth, potentially due to varying countermovement depth across sessions (36), the influence of such variations is considered minimal in this study because the countermovement was standardized.

## 5 Conclusion

A 7-day step taper with 35% training time reduction maintained physical and morphological aspects at the late-season phase towards a national championship in female elite ice hockey players. Moreover, a 2-week training cessation did not lead to decrease in jump height, concentric RFD, maximal strength, or power, but it did result in a decrease in braking RFD and peak force, and muscle mass. This result suggests that while explosive power and strength remain stable, the ability to quickly reduce speed may decline. Furthermore, the decreased GRF during the braking phase generally recovered within 1 week through retraining. It is recommended to perform an evaluation of deceleration ability and focus on training programs aimed at improving it prior to the next season or returning to play.

## Data Availability

The raw data supporting the conclusions of this article will be made available by the authors, without undue reservation.
